# Combination of single quantitative parameters into multiparametric model for ischemia detection is not superior to visual assessment during dobutamine stress echocardiography

**DOI:** 10.1186/s12947-016-0055-6

**Published:** 2016-04-12

**Authors:** Jelena Čelutkienė, Greta Burneikaitė, Linas Petkevičius, Laura Balkevičienė, Aleksandras Laucevičius

**Affiliations:** 1 Clinic of Cardiovascular diseases, Faculty of Medicine, Vilnius University, Vilnius, Lithuania; 2 Centre of Cardiology and Angiology, Vilnius University Hospital, Santariskiu 2, 08661 Vilnius, Lithuania; 3 Faculty of Mathematics and Informatics, Vilnius University, Naugarduko 24, 03225 Vilnius, Lithuania; 4 Centre for Innovative Medicine, Zygimantu 9, 01102 Vilnius, Lithuania

**Keywords:** Coronary artery disease, Dobutamine stress echocardiography, Speckle tracking, Myocardial deformation imaging, Machine learning

## Abstract

**Background:**

To evaluate if the combination of several quantitative parameters into a mathematical model would enhance the detection of myocardial ischemia during dobutamine stress echocardiography (DSE) when compared to conventional wall motion analysis.

**Methods:**

In a prospective study design 151 patients (age 61.8 ± 9.2) in test group and 105 patients (age 64.0 ± 10.6) in validation group were selected and underwent DSE between January 2008 and December 2012. In all patients coronary angiography was performed within 6-8 weeks from DSE, considering at least one stenosis ≥50 % per patient as significant coronary artery disease (CAD). Results of DSE visual assessment and myocardial velocity, strain and strain rate parameters derived from speckle tracking imaging were imported automatically to an originally created software. A mathematical model calculating prognosis of at least one stenosis per patient and stenosis in separate arteries was constructed.

**Results:**

Myocardial ischemia was visually detected in 60 (39.7 %) and in 58 (54.2 %) patients of the test and validation group, respectively. A total of 76 (50.3 %) patients in the test group and 69 patients (65.7 %) in the validation group had ≥50 % coronary stenosis. Sensitivity and specificity of the mathematical model per patient in the test group were 91.6 % and 86.3 % compared to 76.8 % and 89.0 % of the visual assessment, respectively. However, in the validation group the sensitivity, specificity, positive predictive value and negative predictive value dropped down significantly becoming lower to visual assessment.

**Conclusions:**

Myocardial deformation imaging may potentially replace visual assessment with an automated predictive model for stress-induced ischemia detection. However, a multiparametric mathematical model based on quantitative deformation markers did not demonstrate incremental value to visual assessment of wall motion.

## Background

Several studies have proposed a number of quantitative parameters vs. visual assessment for ischemia detection during dobutamine stress echocardiography (DSE) [1–7]. However, most of these quantitative tools have remained in the research laboratory and are not implemented in the routine clinical practice. In a previous report we tried to identify a single powerful quantitative parameter for the prediction of coronary stenosis studying multiple velocity and deformation parameters during DSE but we could not demonstrate that such an approach was better than expert visual wall motion reading [8]. Several previous reports were consistent with our findings showing that visual assessment was equally accurate as quantitative assessment. However, the main limitation of stress echocardiography is related to operator’s experience and a more objective and quantitative approach is needed. The purpose of this study was to evaluate if the combination of several quantitative parameters into a mathematical model would enhance the detection of myocardial ischemia during DSE. The study hypothesis is that a multiparametric approach would provide a sound and effective diagnostic tool.

## Methods

One hundred fifty-one prospectively enrolled consecutive patients in the test group underwent DSE between January 2008 and December 2010 and 105 patients in the validation group underwent DSE between January 2011 and December 2012. Decision for DSE indication was made by consulting cardiologists not involved in the research project, in the course of routine diagnostic workup. DSE was performed for recurrent symptoms in patients with known coronary artery disease (CAD) (*n* = 35 in the test group and *n* = 40 in the validation group) or suspected CAD (*n* = 116 and *n* = 65, respectively). Patients were included in the study if coronary angiography was performed within 6-8 weeks after DSE. Exclusion criteria were: previous myocardial infarction, previous cardiac surgery, non-sinus rhythm, significant valvular disease, left ventricular hypertrophy [9–11], atrial or ventricular arrhythmias, bundle branch block or reduced left ventricular (LV) ejection fraction (EF) <50 %. Beta-blocking medications were discontinued 48 hours, nitrates and other antianginal medications – 24 hours prior to the DSE in all patients. Stress echocardiography was performed on medical therapy in 94 (62 %) patients in the test group and 82 (78 %) in the validation group (calcium-antagonists in 56 and 41, or nitrates in 38 and 21, respectively) and off therapy in 57 (38 %) and 23 (22 %) patients. Informed consent was obtained from all patients before testing, and the study protocol was approved by the Vilnius regional Bioethics committee (Approval No.158200-11-254-58). Stress echo data were collected and analysed by stress echocardiographers not involved in patient care. Hypertension and hypercholesterolemia were defined according to standard definitions [9, 11].

### Dobutamine echocardiography and visual assessment

Each study patient underwent a standard DSE protocol with incremental dobutamine infusion rates 5, 10, 20, 30, and 40 μg/kg/min for 3 minutes each stage under continuous ECG, blood pressure (BP), and echocardiographic monitoring. When no end point was reached, atropine (up to a maximum of 1 mg) was added to the continuing 40 μg/kg/min dobutamine infusion. Non-echocardiographic diagnostic end-points were the following: peak atropine dose; 85 % of target heart rate; development or deterioration of wall-motion abnormalities, severe chest pain and/or diagnostic ST segment changes. The test was also stopped for one of the following reasons: intolerable symptoms, systolic blood pressure increase to >220 mmHg or hypotension, severe arrhythmias.

Transthoracic stress echocardiographic studies were performed with commercially available ultrasound machine (System Vivid 7, GE Healthcare, Horten, Norway) with 1,5 – 4,6 MHz transducer. The long and short axis of the LV from parasternal window, 4- and 2-chamber views from apical window were acquired for comparison in four stages of stress test. Regional wall motion was assessed according to the recommendations of the European Association of Echocardiography dividing LV into 16 myocardial segments. In all studies, segmental wall motion was semiquantitatively graded as follows: normal = 1; hypokinetic, marked reduction of endocardial motion and thickening = 2; akinetic, virtual absence of inward motion and thickening = 3; and dyskinetic, paradoxic wall motion away from the center of the left ventricle in systole = 4. It was considered that in some cases of normal variant basal inferior and basal inferoseptal segments could be scored as hypokinetic. The sum of all segment scores divided by the number of interpretable segments made WMSI. Test positivity was defined as the occurrence of at least one of the following conditions: 1) new dyssynergy in a region with normal resting function (i.e., normokinesis becoming hypo, aki or dyskinetic); 2) worsening of a resting dyssynergy (i.e., a hypokinesia becoming aki or dyskinesia).

### Speckle tracking myocardial imaging

Speckle tracking images (STI) were recorded at baseline and peak dobutamine levels with breath-holding. The frame rate of stored apical 2 and 4-chamber cine-loops for speckle tracking analysis was in the range of 70–90 frames/sec. The loops were stored digitally and analysed off-line using customised software (Echopac PCBT08, GE Healthcare). After manual tracing of endocardium borders in the end-systolic frame of the 2-D images, the software automatically tracked myocardial motion, creating 6 regions of interest in each apical image, with tracking quality labelled as verified or unacceptable. In segments with unacceptable tracking, the observer readjusted the endocardium trace line until a verified tracking was achieved. If this was not attainable, that segment was excluded from analysis. Graphical displays of deformation parameters (reflecting the average value of all of the acoustic markers in each segment) were then automatically generated for 6 segments in each view.

### Measurement of quantitative parameters

Peak longitudinal systolic (S’), diastolic (E’, A’) velocities, time to peak systolic velocity, peak longitudinal and radial systolic, post-systolic and maximal strain, peak longitudinal systolic and diastolic strain rate, radial systolic displacement at rest and during stress were measured using automated vendor-suggested software. Maximal strain coincided with systolic or post-systolic strain whichever was found larger. Parameters of 12 myocardial segments (6 in 4-chamber and 6 in 2-chamber views) were manually approved and automatically exported to Excel tables using commercially available software (Echopac PCBT08, GE, Healthcare). Post-systolic index (PSI) was defined by formula *PSI = peak post-systolic strain - peak systolic strain*. Speckle tracking parameters were automatically imported to multiparametric model integrated in local Access DSE database.

### Coronary angiography

Coronary angiography was performed in all patients of both groups referred to DSE within 6–8 weeks after dobutamine challenge according to the standard Judkins technique adopting femoral or radial approach with Inova2100 (GE Healthcare). Clinical decision to perform coronary angiography was made independently of the study by consulting cardiologists, who were aware of DSE test results for conventional wall motion criteria. At least five views (including two orthogonal views) were acquired for the left and at least two orthogonal views for the right coronary artery, respectively. Additional appropriate projections were obtained in case of superimposition of side branches or foreshortening of the segment of interest. Coronary angiographic data were analysed by 2 experts blinded to the clinical data and the results of DSE. Obstructive CAD was defined as a quantitatively assessed coronary stenosis ≥ 50 %.

### Statistical analysis, multiparametric model construction and implementation

Study variables are presented as mean values ± SD. Interobserver agreement was determined by having two independent investigators measure representative parameters using STI and assess WMSI in 15 randomly selected patients. Intraobserver agreement was determined by having 1 investigator repeat STI measurements and WMSI evaluation in other 15 randomly selected patients 1 month later, while being blinded to the previous measurements. Reproducibility is expressed as the mean percentage difference (value of observer 1 - value of observer 2/mean of the values of observer 1 and 2).

Construction of the underlying statistical model consisted of two steps. The first step was intended for selection of predictive visual assessment and speckle tracking variables. As the amplitude of quantitative covariants depends on the location of the segment in the left ventricle, analysis was performed separately for each segment location. The level of significance was set at 0.05. Simple logistic regression model was fitted for each of the study variable. If a parameter was significant it was included into the set of predictors used in the second step (Table 1). Moreover, for each significant parameter optimal threshold of classification was computed. In the second step all raw values of covariants were replaced by new ones as follows. We denoted optimal logistic regression threshold corresponding to parameter *a* and obtained in the first step, whereas *β*_*α0*_ *, β*_*α1*_ denote the estimated model parameters. For a particular value *x* dependent of parameter *a* calculated in step 1*,* we defined new rescaled logistic regression response (step 2).

**Table 1:** Speckle tracking and visual evaluation parameters constituting multiparametric model

Variable	Segment	Cutoff	Unit
Model for at least one stenosis per patient			
Maximal strain rest	Mid septal	-18.39	%
A’ velocity rest	Mid anterior	-1.83	cm/s
Time to maximal strain rest	Apical septal	394.0	ms
Time to S’ velocity stress	Apical anterior	78.0	ms
Time to S’ velocity stress	Mid anterior	51.0	ms
Systolic strain rate stress	Apical septal	-2.37	s^-1^
Radial systolic displacement rest	Basal lateral	5.42	mm
A’ strain rate stress	Basal lateral	1.96	s^-1^
S’ velocity stress	Basal anterior	6.78	cm/s
Systolic strain rest	Apical septal	-22.43	%
Maximal strain rest	Basal inferior	-20.12	%
Visual	WMSI_stress_-WMSI_rest_	0.13	
Model for LAD			
Systolic strain rest	Apical septal	-11.53	%
Systolic positive strain stress	Basal inferior	0.27	%
Maximal strain rest	Basal inferior	-21.53	%
S’ velocity stress	Basal anterior	8.41	cm/s
E’ velocity stress	Basal septal	-7.2	cm/s
A’ velocity rest	Mid anterior	-1.69	cm/s
Visual	WMSI_stress_-WMSI_rest_	0.13	
Time to maximal strain rest	Apical anterior	353.0	ms
Time to S’ velocity stress	Mid septal	78.0	ms
Time to S’ velocity stress	Apical anterior	120.0	ms
Time to S’ velocity stress	Mid anterior	59.0	ms
A’ strain rate stress	Basal lateral	1.96	s^-1^
Systolic strain rate stress	Apical septal	-2.37	s^-1^
Radial systolic strain stress	Mid septal	8.44	%
Model for LCX			
A’ strain rate stress	Basal lateral	1.70	s^-1^
A’ velocity rest	Mid anterior	-3.42	cm/s
Systolic strain rest	Apical septal	-24.48	%
S’ velocity rest	Apical inferior	3.50	cm/s
E’ velocity stress	Basal septal	-5.51	cm/s
Radial systolic displacement rest	Basal lateral	7.33	mm
Systolic strain rate stress	Apical septal	-2.84	s^-1^
Time to maximal strain rest	Apical anterior	404.0	ms
Time to S’ velocity stress	Apical anterior	56.0	ms
Time to S’ velocity stress	Mid septal	75.0	ms
Radial systolic strain stress	Mid septal	41.90	%
Visual	WMSI_stress_-WMSI_rest_	0.13	
Model for RCA			
Maximal strain rest	Basal inferior	-20.12	%
Systolic strain rest	Apical septal	-22.4	%
S’ velocity rest	Apical inferior	1.38	cm/s
Time to maximal strain rest	Apical anterior	390.0	ms
Time to maximal strain rest	Apical septal	399.0	ms
Time to maximal strain rest	Basal posterior	397.0	ms
Time to S’ velocity stress	Mid anterior	47.0	ms
Systolic positive strain stress	Basal inferior	0.48	%
E’ velocity stress	Basal septal	-2.88	cm/s
Radial systolic displacement rest	Basal lateral	5.42	mm
Visual	WMSI_stress_-WMSI_rest_	0.13	

*LAD* Left ascending artery, *LCX* Left circumflex artery, *RCA* Right coronary artery

$$ r(x)=\left\{\begin{array}{c}\hfill \frac{\varphi \left({\beta}_{0\alpha }+{\beta}_{1\alpha }x\right)-{T}_{\alpha }}{1-{T}_{\alpha }},\mathrm{if}\left({\beta}_{0\alpha }+{\beta}_{1\alpha }x\right)>{T}_{\alpha };\hfill \\ {}\hfill \frac{Ta-\varphi \left({\beta}_{0\alpha }+{\beta}_{1\alpha }x\right)}{T_{\alpha }},\mathrm{if}\left({\beta}_{0\alpha }+{\beta}_{1\alpha }x\right)\le {T}_{\alpha };\hfill \end{array}\right. $$

with *φ*(*y*) = e^y^ */*(1 + e^y^). Now for observation number *i* with particular value of *a* equal to *x*_*i*_ put

$$ {\tilde{x}}_i=\kern0.36em \left\{\begin{array}{c}\hfill 0,\;\mathrm{if}\;{x}_i\;\mathrm{is}\;\mathrm{missing};\hfill \\ {}\hfill r\left({x}_i\right),\;\mathrm{if}\;{x}_i\;\mathrm{is}\;\mathrm{not}\;\mathrm{missing}.\hfill \end{array}\right. $$

**Fig 1:**
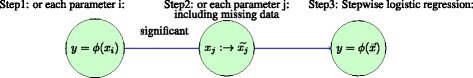
Multiparametric mathematical model construction steps. Step 1 - selections of significant covariants, Step 2 - replacement of missing data, Step 3 - application of logistic regression

Transformation allows use missing values set as zero, otherwise calculated rescale transformation (positive/negative) is more informative in logistic regression. Multiparametric mathematical model construction steps are shown in Fig. 1.

After replacement the data set did not contain missing values and included only those covariants which were selected in the first step. For this full data set stepwise logistic regression was applied. Hence final model could be treated as some kind of voting neural network with unusual fitting method. Described procedure was applied for prognosis of stenosis in separate arteries: left ascending artery (LAD), right coronary artery (RCA), left circumflex artery (LCX) as well as for prognosis of presence of at least one stenosis per patient. Covariants and cutoff included in models are shown in Table 1.

“Test group” provided a dataset used for model construction (151 consecutive patients enrolled between January 2008 and December 2010); “validation group” yielded an independent dataset used to estimate how accurately the model will perform in practice (105 consecutive enrolled between January 2011 and December 2012).

To make model convenient for practitioners a software was incorporated within an existing Access data collection form. Calculations of sensitivity, specificity and accuracy were performed according to standard definitions. The 95 % CIs were calculated and the individual intervals were compared. Differences were considered significant at the 0.05 level when 95 % CI did not overlap.

## Results

### Stress echocardiography

Clinical and echocardiographic characteristics of the study population are reported in Table 2. No major complications occurred during DSE. The 85 % age-predicted maximum heart rate was achieved in 137 (90.7 %) and 94 (89.5 %) test and validation group, respectively. Ischemia was visually detected in 60 (39.7 %) and in 58 (54.2 %) patients of the test and validation group, respectively.

**Table 2:** Clinical characteristics of study groups and DSE hemodynamics

Characteristics	Test group (*n* = 151)	Validation group (*n* = 105)
Age, years	61.8 ± 9.2	64.0 ± 10.6
Male	89 (58.9 %)	66 (62.9 %)
Typical angina	62 (41.1 %)	34 (32.4 %)
Hypertension	141 (93.4 %)	100 (95.2 %)
Hypercholesterolemia	118 (78.1 %)	73 (69.5 %)
Diabetes	29 (19.2 %)	19 (18.1 %)
Smoking	28 (18.5 %)	31 (29.5 %)
MMI, g/m^2^	99.4 ± 17.1	88.4 ± 19.9
EF rest, %	54.5 ± 1.8	53.5 ± 2.9
EF stress, %	59.9 ± 6.6	60.2 ± 6.0
HR rest, beats per min	69.9 ± 11.1	70.4 ± 11.8
HR stress, beats per min	132.4 ± 10.9	130.2 ± 14.2
ECG changes during stress	77 (51.0 %)	38 (36.2 %)
Chest pain during stress	87 (57.6 %)	57 (54.3 %)
WMSI rest	1.02 ± 0.04	1.05 ± 0.10
WMSI stress	1.21 ± 0.23	1.18 ± 0.20

*MMI* Myocardial mass index, *EF* Ejection fraction, *HR* Heart rate, *BP* Blood pressure, *ECG* Electrocardiogram, *WMSI* Wall motion score index

### Feasibility and reproducibility of quantitative data

After exclusion of poorly visualized segments, the stored data of 1466 (97.1 %) and 1017 (96.9 %) myocardial segments in the test and validation group, respectively, were finally analysed. The prevalence of uninterpretable signals due to inadequate tracking in the segments included in the final analysis was found to be 2.1 % and 2.5 % at rest and 5 % and 5.2 % during stress in the test and validation group, respectively.

The mean percentage differences of inter- and intraobserver measurements of velocity, strain, strain rate and WMSI are summarized in Table 3.

**Table 3:** Reproducibility of visual and quantitative methods (mean percentage difference)

	Visual		Speckle tracking imaging					
	*WMSI*		*Velocities*		*Strain*		*Strain rate*	
	Rest	Stress	Rest	Stress	Rest	Stress	Rest	Stress
*Interobserver*	0.031	0.035	0.029	0.031	0.075	0.077	0.007	0.009
*Intraobserver*	0.017	0.028	0.026	0. 037	0.097	0.109	0.010	0.016

*WMSI* Wall motion score index

### Angiographic results

A total of 76 (50.3 %) patients in the test group and 69 patients (65.7 %) in the validation group had ≥50 % coronary stenosis, see Table 4.

**Table 4:** Extent of CAD in test and validation groups

	Test group (*N* = 151)	Validation group (*N* = 105)
No stenosis	75 (49.7 %)	36 (34.3 %)
1 vessel	32 (21.2 %)	25 (23.8 %)
2 vessels	23 (15.2 %)	22 (20.95 %)
3 vessels	21 (13.9 %)	22 (20.95 %)
>50 % stenosis	20 (13.2 %)	15 (14.3 %)
>70 % stenosis	21 (13.9 %)	22 (21.0 %)
>90 % stenosis	35 (23.2 %)	32 (30.5 %)

### Diagnostic accuracy of multiparametric model and visual wall motion analysis

**Fig. 2:**
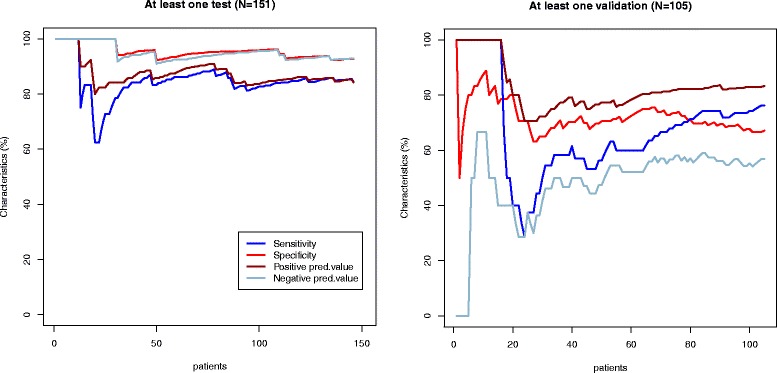
Diagnostic performance of at least one stenosis per patient model. The sensitivity (blue line), specificity (red line), positive predictive value (brown line) and negative predictive value (light blue line) curves are depicted in the test (Part 1) and validation (Part 2) groups

**Fig. 3:**
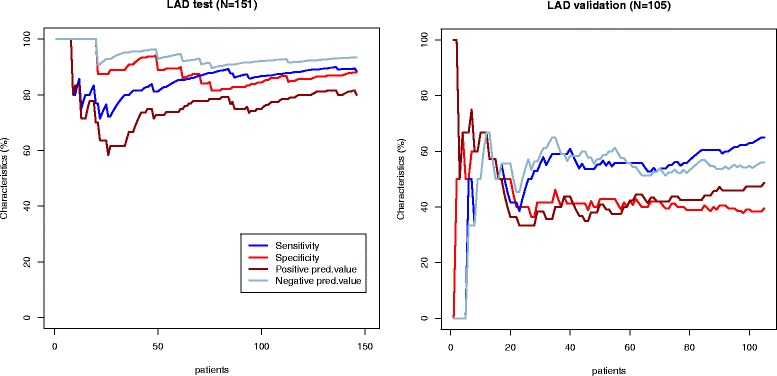
Diagnostic performance of LAD model. The sensitivity (blue line), specificity (red line), positive predictive value (brown line) and negative predictive value (light blue line) curves are depicted in the test (Part 1) and validation (Part 2) groups

**Fig. 4:**
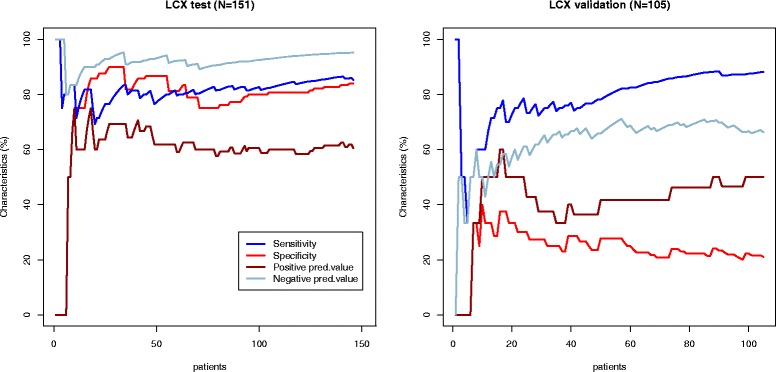
Diagnostic performance of LCX model. The sensitivity (blue line), specificity (red line), positive predictive value (brown line) and negative predictive value (light blue line) curves are depicted in the test (Part 1) and validation (Part 2) groups

**Fig. 5:**
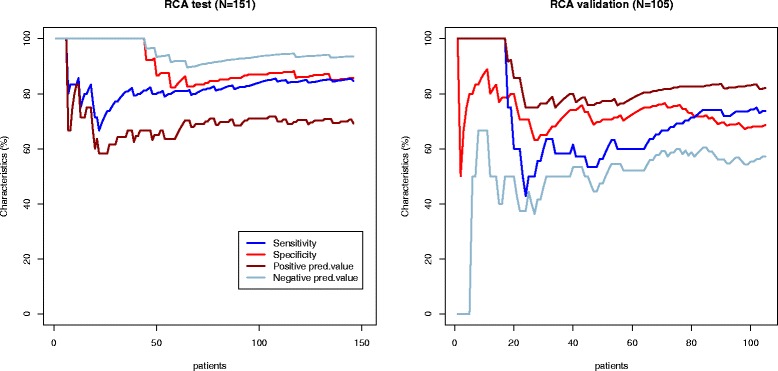
Diagnostic performance of RCA model. The sensitivity (blue line), specificity (red line), positive predictive value (brown line) and negative predictive value (light blue line) curves are depicted in the test (Part 1) and validation (Part 2) groups

Diagnostic performance of models created per patient and per vessel in the test group appeared to be superior to visual assessment (Table 5, Figs. 2, 3, 4 and 5). Sensitivity and specificity of the model per patient in the test group were 91.6 % and 86.3 % compared to 76.8 % and 89.0 % of visual assessment, respectively. However, when we applied the same models in the new validation group, sensitivity, specificity, positive predictive value and negative predictive value were significantly reduced and became lower to visual evaluation (Table 5, Figs. 2, 3, 4 and 5).

**Table 5:** Performance of multiparametric model and visual wall motion analysis in the test and validation groups

	Group	Sensitivity, % (95 % CI)	Specificity, % (95 % CI)	Positive predictive value, % (95 % CI)	Negative predictive value, % (95 % CI)
Visual wall motion analysis	Test	76.8 (65.6; 85.2)	89.0 (80.4; 94.1)	85.5 (74.7; 92.2)	82.0 (72.8; 88.6)
	Validation	75.8 (64.2; 84.5)	74.4 (58.9; 85.4)	83.3 (71.9; 90.7)	64.4 (49.8; 76.7)
Model for at least one stenosis for patient	Test	91.6 (82.8; 96.1)	86.3 (77.0; 92.2)	85.5 (75.9; 91.7)	92.0 (83.6; 96.3)
	Validation	66.7 (55.9; 76.7)	77.8 (61.9 ;88.3)	85.2 (73.4; 92.3)	54.9 (41.4; 67.7)
LAD model	Test	90.6 (79.8; 95.9)	92.9 (86.0; 96.5)	87.3 (76.0; 93.7)	94.8 (88.4; 97.8)
	Validation	40.4 (28.2; 53.9)	66.0 (52.6; 77.3)	53.9 (38.5; 68.4)	53.0 (41.2; 64.6)
LCX model	Test	85.6 (70.6; 93.7)	94.0 (88.1; 97.1)	81.0 (65.8; 90.5)	95.6 (90.1; 98.1)
	Validation	20.5 (10.8; 35.5)	87.8 (77.9; 93.7)	50.0 (28.0;72.0)	65.2 (54.8; 74.3)
RCA model	Test	85.4 (72.8; 92.7)	92.2 (85.4; 96.0)	83.7 (71.0; 91.5)	93.1 (86.5; 96.6)
	Validation	45.5 (31.7;59.9)	78.7 (66.9; 87.1)	60.6 (43.7; 75.3)	66.7 (55.2; 76.5)

*LAD* Left ascending artery, *LCX* Left circumflex artery, *RCA* Right coronary artery, *CI* Confidence interval

## Discussion

This study represents further consecutive step in attempts to implement quantitative tools in the detection of myocardial ischemia during stress echocardiography. It is based on the number of previous investigations showing the significant links of several myocardial motion and deformation markers with induced ischemia [1–8, 12–15].

However, in the vast majority of publications the diagnostic accuracy of the quantitative markers is demonstrated to be lower or only comparable to the visual assessment of stress echocardiography [1, 2, 8, 12–14]. Current lack of evidence on effective application of quantitative methods in routine practice is reflected in recommendation documents and consensus statement of EAE and ASE [3, 15, 16].

Prior research [1, 4–8] was mostly focused on single parameters, segment-specific or averaged for all myocardial segments that carry only fragmental information of regionally impaired myocardial mechanics. Therefore, we hypothesized that a multiparametric model, including a substantial list of informative quantitative parameters, would demonstrate better performance than separate markers alone or visual DSE assessment.

Theoretically, such mathematical model could better reflect the complicated nature of the biological phenomenon of regional ischemia and incorporate relevant interdependencies between physiologically different parameters. The feasibility was considered as one of the main requirements to the quantitative tool for routine clinical practice, therefore automatically obtainable data of speckle tracking were chosen for model creation.

In parallel with previous studies the set of predictive markers included blunted response of systolic velocity, prolonged time to peak systolic velocity [1, 12, 14], decreased E’ wave velocity [4, 5, 17], longitudinal and radial systolic, post-systolic and maximal strain, post-systolic index [6, 7, 13], systolic and diastolic longitudinal and radial strain rate, radial systolic displacement. In this study we followed the methodology of defining thresholds separately for each evaluated myocardial segment, taking into account known base-to-apex and wall-to-wall differences of myocardial velocity and strain/strain rate [10, 18, 19]. Model user should only approve peak velocity, strain, strain rate and displacement values in the commercially available 2D strain analysis software and then export data set through Excel tables to the constructed system. Originally created classifier was incorporated in daily used Access database of DSE. Visual assessment data were eligible for automatic import, and changes in scores of selected segments entered the model, too. Of note, the lower sensitivity of visual assessment in the present and some previous reports [14] reflects the limitations of subjective interpretation of regional wall motion and justifies the search of quantitative tools.

The constructed mathematical analysis tool represents a kind of machine learning methodology, namely a type of neural network with unusual fitting method. Machine learning technology is currently well suited for analysing medical data, and in particular there is a lot of work done in medical diagnosis in small specialized diagnostic problems [20–22]. This system provides a possibility to handle an unusually large amount of data in a relatively short period of time. The created classifier automatically made the prognosis of significant coronary stenosis, and in the test group it demonstrated promising results. However, the attempt to validate the model in the similar population of consecutive patients gave disappointing results.

Failure of model validation recalls the shortcomings of single quantitative markers, having rather modest predictive ability of significant coronary stenosis (AUCs 0.60-0.72) [8, 14]. Limited value of distinguished indices could be largely attributed to known technical challenges of quantitative imaging: potentially inadequate spatial and temporal resolution, higher speckle decorrelation between subsequent frames at higher heart rates, noise and artefacts [23]. Similar to our findings, considerable inter- and intra-observer variability of 7-12 % is reported for speckle tracking technology [8, 24–26]. Possibly, mutual interaction of ischemic and non-ischemic segments and load-dependency of deformation parameters may diminish the differences between markers of these two groups [26, 27]. Furthermore, previously demonstrated significant heterogeneity of left ventricular wall thickening during dobutamine stress even in the absence of CAD may contribute to insufficient accuracy of created model [28].

### Study limitations

In this study coronary angiography was used as the reference method. However, relationship between stenosis severity and physiological reduction of coronary flow is quite variable. Angiographic coronary stenosis does not always reflect the potential alteration in the regional myocardial perfusion.

Acquisition of quantitative parameters was based on the commercially available software, therefore relying on implemented methods of noise and artefacts handling. Creating a multiparametric mathematical model, the main challenges remain related to identifying the best methodology for data transformation and critical clinical data. Finally, there is need to understand how to deal with missing data. The study model was constructed on a relatively small data base with possible over fitting.

## Conclusions

Myocardial deformation imaging provides potential for creation of automated predictive model for stress-induced ischemia detection. However, a multiparametric mathematical model based on quantitative deformation markers did not demonstrate incremental value to visual assessment of wall motion.

## Abbreviations

CAD: Coronary artery disease
DSE: Dobutamine stress echocardiography
ECG: Electrocardiogram
EF: Ejection fraction
LAD: Left ascending artery
LCX: Left circumflex artery
LV: Left ventricular
RCA: Right coronary artery
STI: Speckle tracking imag
WMSI: Wall motion score index
